# Pluripotent Stem Cells as a Preclinical Cellular Model for Studying Hereditary Spastic Paraplegias

**DOI:** 10.3390/ijms25052615

**Published:** 2024-02-23

**Authors:** Devid Damiani, Matteo Baggiani, Stefania Della Vecchia, Valentina Naef, Filippo Maria Santorelli

**Affiliations:** 1 Molecular Medicine for Neurodegenerative and Neuromuscular Diseases Unit, IRCCS Fondazione Stella Maris, Via dei Giacinti 2, 56128 Pisa, Italy; matteo.baggiani@fsm.unipi.it (M.B.); stefania.dellavecchia@fsm.unipi.it (S.D.V.); valentina.naef@fsm.unipi.it (V.N.); filippo.santorelli@fsm.unipi.it (F.M.S.); 2 Department of Neurosciences, Psychology, Drug Research and Child Health (NEUROFARBA), University of Florence, Viale Pieraccini, 6, 50139 Florence, Italy

**Keywords:** hereditary spastic paraplegia, induced pluripotent stem cells, pluripotent cells, preclinical model, cellular model

## Abstract

Hereditary spastic paraplegias (HSPs) comprise a family of degenerative diseases mostly hitting descending axons of corticospinal neurons. Depending on the gene and mutation involved, the disease could present as a pure form with limb spasticity, or a complex form associated with cerebellar and/or cortical signs such as ataxia, dysarthria, epilepsy, and intellectual disability. The progressive nature of HSPs invariably leads patients to require walking canes or wheelchairs over time. Despite several attempts to ameliorate the life quality of patients that have been tested, current therapeutical approaches are just symptomatic, as no cure is available. Progress in research in the last two decades has identified a vast number of genes involved in HSP etiology, using cellular and animal models generated on purpose. Although unanimously considered invaluable tools for basic research, those systems are rarely predictive for the establishment of a therapeutic approach. The advent of induced pluripotent stem (iPS) cells allowed instead the direct study of morphological and molecular properties of the patient’s affected neurons generated upon in vitro differentiation. In this review, we revisited all the present literature recently published regarding the use of iPS cells to differentiate HSP patient-specific neurons. Most studies have defined patient-derived neurons as a reliable model to faithfully mimic HSP in vitro, discovering original findings through immunological and –omics approaches, and providing a platform to screen novel or repurposed drugs. Thereby, one of the biggest hopes of current HSP research regards the use of patient-derived iPS cells to expand basic knowledge on the disease, while simultaneously establishing new therapeutic treatments for both generalized and personalized approaches in daily medical practice.

## 1. Introduction

HSPs are a heterogeneous group of rare neurological diseases caused by mutations in a variety of genes. More than 90 SPG (for “SPastic parapleGia”) loci/genes have been identified [1], and the number will continue to grow as long as new sequence technologies are used in clinical laboratory practice. HSPs can follow any hereditary pattern, namely autosomal dominant (AD), autosomal recessive (AR), X-linked recessive (XLR), and mitochondrial modalities [2]. Sporadic HSPs due to true de novo mutations, genealogical censure, non-penetrant AD mutations, or singletons in unrecognized AR kindreds, are also common [3]. The estimated prevalence is 3–10/100,000 in most populations [4], with pure forms more prevalent in Northern Europe [5] and complicated forms (generally AR-transmitted [6]) more prevalent in regions where consanguinity is more common.

HSPs are neurodegenerative disorders primarily involving the longest axons of the central nervous system (CNS). Although the longest sensory neuron axons can also be affected, the descending corticospinal tract is the one that is mainly damaged, impairing transmission from the upper motor neuron cell bodies in the motor cortex to the lower α1-motor neurons that control the leg muscles [7,8,9]. Harding classified HSPs into pure and complicated types [10], with pure forms presenting progressive lower-extremity spastic weakness, urinary disturbance, and mild diminution of vibration sensation. In complicated forms, numerous other clinical manifestations can be found, such as ataxia, extrapyramidal signs, dementia, psychiatric impairment, epilepsy, peripheral neuropathy, and ocular involvement. AD-HSPs are generally pure, and SPG4 is the most common genetic variant [11], followed by SPG3A, SPG31, and SPG10 [12]. AR-HSPs are mostly complicated and more heterogeneous than AD-HSPs [13]. Among them, the most frequent is SPG11, followed by SPG7, SPG5, and SPG15 [12]. In the last decades, numerous efforts have been made to define new classifications and understand molecular mechanisms and pathways involved in HSP [2,3]. Mutations in SPG genes cause cellular organelle morphological and functional impairment in corticospinal neurons and axonal fascicles starting from them. To summarize, proper organization of the endoplasmic reticulum (ER) is disturbed in SPG3A, SPG4 (see related paragraphs below), SPG12, and SPG31, while modulation of lipid metabolism and droplet formation is perturbed in SPG20 and SPG43. In addition, lysosomal formation and regeneration in the endocytic process are hampered in SPG11 and SPG15, mitochondrial failure occurs in SPG7, SPG13, SPG20, and SPG74, and issues in TGN (Trans-Golgi Network) are caused by mutations in gene encoding AP4 complex components in SPG48, SPG50, SPG51, and SPG52. Finally, corticospinal tract degeneration itself could result from defects in axonal elongation pathways, as occurring in SPG1, or myelination errors, as seen in SPG44 and SPG47 (see Martinuzzi et al., 2021 [14] and references therein).

Despite all these efforts, a cure for HSP, or even reliably effective treatment, remains undefined. Over time, numerous experimental models have been used to study HSPs. The use of in vivo HSP models, however, has often been unsuccessful; with the exception of a few selected cases, most SPG mouse models do not develop defects in motor function and have comparable survival respect to control siblings. In the same way, models such as zebrafish (*Danio rerio*) can be useful for understanding the functional connection between a human pathological mutation and disease [15,16], although they have important limits, such as the absence of corticospinal tracts [17]. Limitations in the use of animal models in both phenotypic characterization and the discovery of candidate drugs translatable to human diseases [18] have raised questions about which models should be used to best represent the biology of motor neuronopathies (Table 1).

The study of neurodegenerative diseases in vitro has typically used primary neuronal cultures derived from embryonic or adult nervous tissue. However, the obviously limited availability of patient-derived biopsies is rarely compatible with the average sample size needed for setting up experimental conditions strictly required to generate robust data. In this sense, the rise of iPS cells, combined with the development of protocols aimed to differentiate culture layers of neurons specific to different brain regions from mouse and human pluripotent cells [78,79,80,81,82,83,84], has provided researchers with a way to possibly overcome these known hurdles. However, the extreme cellular diversity, combined with the complex relationship between neuronal and non-neuronal cells, and the highest level of interconnection between different neuronal cell types among different brain locations, rendered the classical bidimensional culture much too reductionistic. For this reason, technological advancements in cell biology allowed the generation of tridimensional neuronal aggregates, starting from pluripotent murine and human cells. The use of these structures, termed organoids, offered a new level of complexity, with the goal of reproducing consistently faithful models of brain tissues, [85,86] and developing new hopes in personalized medicine [87].

In the last decade, basic and preclinical research in spastic paraplegia regularly profited from the use of patient-derived iPS cells to generate and study neurons carrying clinically relevant mutations (see Table 2 for a detailed summary related to the SPG forms discussed here and Table 3 for relative genomic and RNA information). In this review, we will discuss the current knowledge in the field of HSP using iPS cells as a research model.

## 2. HSP-Related iPS Cell Lines

Herein, we summarize the state-of-the-art in the pertinent literature for the diverse forms of HSP in relation to breakthroughs obtained by using pluripotent stem cells.

### 2.1. SPG3A

**Figure 1:**
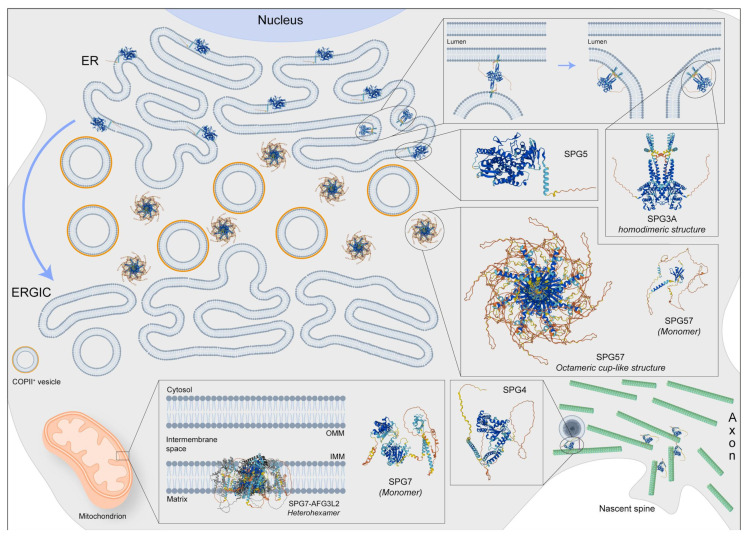
Representative image regarding the physiological localization and possible 3D structure of five SPG proteins: SPG3, SPG4, SPG5, SPG7, and SPG57. SPG3 is also involved in membrane fusion at the endoplasmic reticulum (ER), while SPG4 has several functions including microtubule dynamic in spine formation and regulation of vesicle transport. Moreover, the SGP5 protein should be localized at the ER but its function is poorly known in the brain. Instead, the SPG7 protein, forming the heterohexameric complex with AFG3L2, is an m-AAA protease, also regulating the degradation of damaged proteins, the synthesis respiratory chain, and the assembly of mitochondrial ribosomes. Finally, the SPG57 octameric structure is located between ER and ERGIC (ER-Golgi intermediate compartment) membranes, facilitating COPII vesicle export from the ER and retaining cargo-containing COPII vesicles at the ER/ERGIC interface. Protein 3D structures have been generated with Alphafold [118,119].

Spastic paraplegia 3A (SPG3A) represents the most common cause of early-onset AD-HSP, with an average onset at the age of four years [111]. The disease generally occurs in a pure form with slow progressive spasticity and weakness in the lower limbs [112]. However, it can also manifest as a complicated form, in which spasticity is associated with peripheral neuropathy and distal amyotrophy because of lower motor neuron involvement [113]. Magnetic resonance imaging (MRI) showed significant alterations in the transverse areas of the cervical and thoracic spinal cord [114] and the presence of a thin corpus callosum (TCC) [115]. SPG3A is caused by mutations in the *ATL-1* gene, encoding the protein Atlastin-1, localized in the ER where it remodels lipid membranes [116], mediating homotypic fusion of tubules to form polygonal ER networks [117] (Figure 1).

Disease-causing variants not only lead to altered vesicle trafficking between ER and Golgi but also to altered Golgi morphogenesis [120]. Although mutations in *ATL-1* are predominantly missense, in-frame deletions have also been reported [3]. Together with Spastin, REEP1, and Reticulon 2, mutations in Atlastin-1 are responsible for abnormal membrane trafficking and abnormal organelle shaping; in fact, these proteins share similar hairpin loops that control ER membrane shaping.

**Figure 2:**
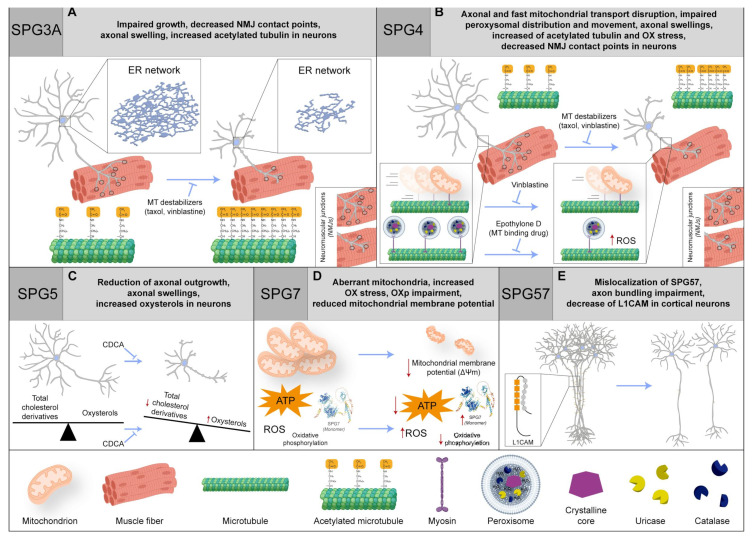
(**A**) In neurons, SPG3 impairment hampers the growth and NMJ contact points, causes axonal swelling, and increases the acetylated tubulin. Intriguingly, there is a recovery with MT-binding agents (such as taxol and vinblastine). (**B**) In SPG4 patients, the axonal transport is disrupted, coupled with an impairment in fast mitochondrial transport and peroxisome distribution and movement. The phenotype is rescued by vinblastine or epothilone D (MT-binding drug). Moreover, in neurons, axonal swelling is reported, and there is an increase in acetylated tubulin and oxidative (OX) stress, together with a decrease in NMJ contact points. Even in this case, a recovery with MT-binding agents (such as taxol and vinblastine) is found. (**C**) In SPG5 neurons, the axonal swelling phenomenon is found as well as an increase in oxysterols but not in total cholesterol derivatives and axonal outgrowth reduction. Importantly, chenodeoxycholic acid (CDCA) treatment can rescue the phenotype. (**D**) SPG7 patients with compound heterozygosity display an increased SPG7 quantity and OX stress, OX phosphorylation (OXp) impairment, and aberrant mitochondria, with a reduction in mitochondrial membrane potential and ATP production. (**E**) In SPG57 cortical neurons, there is an impairment of axon bundling, an L1CAM decrease, and SPG57 protein mislocalization. Red arrows indicate a dysregulation of biochemical pathways, unbalance of protein levels or changes in molecular specie concentrations. Protein 3D structures have been generated with Alphafold [118,119].

In an effort to unveil the cellular and molecular bases of neuronal degeneration, several model systems have been generated. In mice, *Atl-1* mRNA has been shown to be enriched in layer V of the cerebral cortex during early development [21] (Table 1). There, Atlastin-1 is supposed to play an important role in the morphogenesis of dendrites [20], as knockdown in primary cortical neurons causes impairment of axonal growth and branching defects [19] (Figure 2A). Using episomal-mediated reprogramming, several pluripotent stem cell clones were derived from a 2-year-old female patient affected by pure HSP and carrying the heterozygous Pro342Ser mutation in *ATL-1* [90], in a residue that is fully conserved across eukaryotes. Although the mutation did not abrogate protein translation and localization in patients’ fibroblasts, the mutated proline resides in a linker that is crucial for a conformational switch of Atlastin-1, strictly required for both the ER tubule fusion process and full GTPase activity. Similar to what had previously been observed in patient-derived fibroblasts, the Pro342Ser mutation did not affect Atlastin-1 protein levels in iPS-derived forebrain glutamatergic neurons. However, consistent with what was previously observed in the zebrafish model [22,23] (Table 1), SPG3A cortical neurons derived from two different iPS clones displayed a significative reduction in the average length of axonal branches, implying an impairment of axonal outgrowth. In addition, in order to study the efficiency of anterograde and retrograde transport, mitochondrial movements across axonal compartments were monitored in iPS-derived mature (12 weeks old) cortical neurons by means of MitoTracker CMX Ros live-cell imaging. No significant differences were detected between mitochondrial transport velocities from control and SPG3A neurons, in both anterograde and retrograde directions, suggesting that the activity of molecular motors was unaffected by *ATL-1* mutations. Despite this, a substantial decrease in anterograde motile events was detected in the SPG3A background, with only 50% of mitochondria actively moving in axons from mutant cells. Finally, as Atlastin-1 is an important binding partner of the SPG4 protein Spastin, a microtubule-severing AAA ATPase, researchers hypothesized that a microtubule-destabilizing approach could be beneficial in the SPG3A background. That said, administration of taxol and vinblastine was performed during SPG3A neuronal differentiation (Figure 2A), resulting in almost complete rescue of the axonal outgrowth phenotype in *ATL-1* mutant cells and confirming the involvement of microtubule dynamics in SPG3A etiopathology.

In another more recent study [91], iPS cells derived from three different SPG3A patients carrying heterozygous mutations in *ATL-1* were differentiated in lower motoneurons following established protocols [121], generating a homogeneous population of cells expressing both the neuronal NEFH (Neurofilament Heavy) and ISL1 (Islet-1) motoneuron markers. Despite differentiation not being perturbed in the presence of mutated Atlastin-1, with no obviously detectable differences between the control and SPG3A cell lines, the immunostaining of patient-derived neurons revealed a significant increase in swelling in the axonal compartment, accompanied by a relative accumulation of acetylated tubulin (labeling stable microtubules; Figure 2A), compared to motoneurons derived from healthy iPSC lines. In addition, iPSCs were also differentiated in skeletal muscular tissue and combined with the cognate motoneurons in microfluidic devices, following established procedures [122] aimed at achieving in vitro formation of neuromuscular junctions (NMJs). The interaction of neurites with myotubes was confirmed in all cases, but the typical proportion of single- and multiple points of contact present in control NMJs was disrupted in SPG3A cell lines, with an increase in single-points and a decrease in multiple points of contact. Accordingly, a decrease in the expression for genes encoding acetylcholine receptor subunits (i.e., *CHRNB1*, *CHRNA1*, and *CHRNG*) was detected in SPG3A-mutated co-cultures, whereas no significant differences were found in genes related to inflammation (such as *TNFa*, *IL6*, *IL1R*, and IL10) or autophagy (e.g., *BECN1*, *MAP1LC3A*, *MAP1LC3B*, *MAP1LC3C*, and *SQSTM1*). Together, these experiments confirmed improper NMJ development in SPG3A disease and confirmed the usefulness of iPS cells for basic and preclinical research in this field.

### 2.2. SPG4

Spastic paraplegia 4 (SPG4; also known as SPAST-HSP) is the most frequent form in both familial and sporadic AD-HSPs [3,11]. Generally, patients have a pure phenotype with onset in teen years or early adulthood [123], but complicated features are also possible in SPG4 presentations [124], even with intra- and inter-familial variability [125,126]. Brain MRI is often normal or presenting with mild vermis atrophy, TCC, subtle white matter changes, and/or cerebellar atrophy [114]. Prominent spinal cord atrophy can also be retrieved at spinal cord MRI.

Patients affected by SPG4 carry mutations in *SPAST*. This gene codes for Spastin, a 616 amino acid (67.2 kDa) protein belonging to the AAA (ATPases associated with diverse cellular activities) group of proteins, involved in cell cycle regulation, protein degradation, organelle biogenesis, and vesicle-mediated functions [127] (Figure 1). Spastin has two isoforms, M1 and M87, differing in the presence or absence of 86 N-terminal amino acids, and each of them can be encoded by different isoforms lacking exon 4 (M1ΔEx4 and M87ΔEx4, respectively [128]). The protein is involved in microtubule disassembly [129] and enriched in the distal axon of corticospinal motoneurons [130], the nervous structure typically undergoing degeneration in HSP patients. Most pathogenic mutations involve the AAA domain and act through a mechanism of loss of function [131], predominantly causing axonal transport dysfunction [3] (Figure 2B). However, haploinsufficiency, or a combination of loss and gain of function, has also been invoked as a mutation-dependent mechanism for SPG4 [33].

To unravel the mechanisms underpinning the etiopathogenesis in SPG4, one of the first approaches consisted of deriving neural progenitor cells as floating neurospheres from biopsies of the SPG4 patient’s olfactory mucosa, successively propagated in adherent cultures as ONS (Olfactory NeuroSphere-derived) cells [92]. Similar models have been successfully used in other contexts to reveal significant and novel cellular aspects of genetically uncharacterized diseases such as schizophrenia and sporadic Parkinson’s disease. ONS cells derived from 9 patients and 10 healthy controls were compared via different approaches, such as flow cytometry, microarray-based gene expression profiling, and analysis of protein expression and cellular function. Strikingly, patient-derived cells gave homogeneous results for all experiments performed, irrespective of the type of *SPAST* mutation. In line with this, protein levels of both Spastin isoforms showed a 50% reduction in *SPAST*-mutated cells compared to wild type counterparts. In particular, gene expression was dysregulated, with differential expression for 57% of the genes analyzed. In line with the known function of the Spastin protein, cluster analysis of differentially expressed genes (DEGs) showed transcriptional regulation of genes regulating microtubule polymerization, stabilization, bundling, and organization, such as tubulins, kinesins, and Stathmin (encoded by *STMN1* gene), most of them being overexpressed in *SPAST*-mutated cells. Consistently, the Stathmin protein level was increased by 50%, and this could partly explain why acetylated tubulin (a marker of stable microtubules), was decreased in ONS cells, contrary to expectations based on Spastin function (Figure 2B).

The same SPG4 patient- and healthy donor-derived ONS cell lines were used in a more recent study [95], where cells were differentiated into multipolar and bipolar cells containing axonal-like processes similar to neurites. As impairment of axonal transport is deemed a possible mechanism in SPG4 pathogenesis, the distribution of peroxisomes along processes was studied by staining neuron-like cells to label peroxisomal membrane protein PEX14. Differentiated SPG4-derived cells exhibited a substantial reduction in the peroxisome number in axon-like processes compared to control-derived neurons (58.06 vs. 80.4, respectively). In addition, differentiated ONS cells were transduced with a construct live-cell GFP peroxisome probe (CellLight Peroxisome-GFP BacMam 2.0, Thermo Scientific, Milan, Italy) to perform live imaging of moving peroxisomes along axon-like processes. Despite the overall motility behavior of single peroxisomes being conserved, suggesting normal interactions with microtubules and molecular motors, the percentage of fast-moving organelles was significantly reduced (2.3% vs. 10%) in SPG4 compared to the control population. In addition, axonal movements of peroxisomes shifted from retrograde to anterograde, generating a diverse cellular distribution that well correlates with increased sensitivity of *SPAST* mutant cells to physiological and peroxide-induced oxidative stress, as testified by increased immunoreactivity for the free radical compound 4-hydroxy-2-nonenal (4-HNE). Oxidative stress was completely rescued by treatment with the microtubule-stabilizing drug epothilone D (Figure 2B), corroborating cytoskeletal involvement in this phenotype, and raising the hypothesis that the impairment of peroxisome transport could create local domains of oxidative stress, ultimately leading to neuronal toxicity and cell death [132].

In another study [94], dermal fibroblasts taken from two patients carrying a heterozygous *SPAST* nonsense mutation served to generate iPS cells. Control fibroblast lines were derived from age-matched healthy controls with no history of movement disorder or neurologic disease. Spastin isoforms were evaluated in fibroblasts, iPSCs, NPCs, and primary astrocytes. While the two major isoforms present in every cell type were M87 and M87ΔEx4, the full-length M1 isoform was restricted exclusively to NPCs and neuronal cultures. Notably, the expression level of Spastin was increased in neural lineage. Spastin isoforms were overall downregulated in SPG4 iPS-derived neurons by nonsense-mediated decay, with a 34% and 40% decrease for M1 and M87 isoforms, respectively, corresponding with an overall decrease of almost 50% for the Spastin protein level. In addition, while the soma size did not change in the different experimental groups, SPG4 neurons presented significantly fewer primary neurites and reduced the total neuritic length, accompanied by a significant decrease in the number of branching points. Scholl analysis also confirmed the reduction in neuritic complexity in SPG4 neurons, especially in regions proximal to the cell soma. In addition, it is worth noting that the enlargement of axons for the focal accumulation of organelles, a condition termed axonal swelling [30,31], represents a common pathologic hallmark observed in the post-mortem spinal cord of SPG4 patients (Figure 2B). Differentiated neurons showed similar phenotypes, displaying disorganized and interrupted microtubules with abundant swellings and the accumulation of mitochondria. In spite of this, no differences were observed in terms of the total tubulin detyrosination or acetylation levels. A possible compensative mechanism in SPG4 cells could be represented by the observed upregulation of p60 Katanin, a protein with a similar microtubule-severing function as Spastin. As alteration of axonal transport is common in HSP, researchers investigated whether the altered morphology of the SPG4 neurites could also have an effect on organelle transport along axons. To do this, neurons were grown on microfluidic chambers, in a way that axonal projections could pass from one culture chamber to the other by crossing through the device’s microchannels [94]. Differentiated neurons were infected with lentiviruses expressing Mito-DsRed in order to visualize moving mitochondria in neurites via imaging of the microchannels. In this study, the amount of actively transported mitochondria was unchanged, similar to the speed of transported organelles. Despite this, imbalances in the anterograde and retrograde movements were identified (Figure 2B).

A similar study [93], reprogramming iPSC clones from patient fibroblasts carrying splice-site mutations, showed an increased abundance of acetylated tubulin upon differentiation in dorsal telencephalic neurons, confirming the hypothesis that the reduction in the Spastin level correlates with an increased quantity of stabilized microtubules. Most importantly, acetylated tubulin staining detected a substantial increase in swellings in axons of SPG4-derived neurons, especially in cells displaying longer neurites. As the accumulation of mitochondria (stained with MitoTracker Red CMXRos, Thermo Scientific, Milan, Italy) in swellings was also detected, it was possible to observe impaired fast axonal transport in 8-week-old SPG4-derived neurons, indicating that the impairment of retrograde axonal transport could be a key player in SPG4 pathogenesis. Finally, the link between SPG4 with increased microtubule stability was confirmed via rescuing the neuronal phenotype by treating it with microtubule destabilizing drugs. In particular, the administration of vinblastine at nanomolar concentrations significantly reduced the number of axonal swellings in 8-week-old SPG4-derived or *SPAST* knockdown neurons [93], definitely confirming the involvement of microtubule impairment in SPG4 pathogenesis (Figure 2B). To summarize, similar to SPG3, the use of iPS cells successfully achieved modeling for SPG4 disease, faithfully reproducing the cellular hallmark of patient neurons. It will be interesting in the future to see possible applications of microtubule drugs, such as taxol and vinblastine in both SPG3A and SPG4 patients, even considering the known side effects of these popular drugs.

### 2.3. SPG5

The regulation of cholesterol and biliary acid metabolism is crucial for the development and maintenance of central nervous systems, and defects in cholesterol-associated pathways could potentially lead to neurological diseases, such as amyotrophic lateral sclerosis (ALS) [133,134]. For instance, defects in cholesterol derivative degradation are associated with severe conditions, such as HSP and cerebrotendinous xanthomatosis (CTX). In the acidic pathway of degradation, cholesterol-containing compounds are oxidized at side chains, producing oxysterols (25- or 27-hydroxycholesterol, 25- or 27-OHC), which are in turn α-hydroxylated by the cytochrome P450 Family 7 Subfamily B Member 1 (CYP7B1) [135] (Figure 1). The accumulation of oxysterols, due to biallelic loss of function mutations in CYP7B1, causes neuronal toxicity and impairs synaptic function [136,137] associated with spastic paraplegia 5 (SPG5) [138]. SPG5 is a rare subtype of AR-HSP that typically manifests with periventricular and subcortical white matter lesions on brain MRI [139]. To shed light on the mechanism of the corticospinal axonal degeneration characteristic of human disease, skin fibroblasts derived from healthy donors and SPG5 patients were reprogrammed into iPS cells [96]. SPG5 pluripotent cells were then differentiated in cortical tissue using established protocols, starting from stem cell aggregates, and passing through a neuroepithelial rosette stage to the final glutamatergic forebrain specification [140,141]. Consistent with the known metabolic defect observed in the absence of the CYP7B1 protein, the total cholesterol amount was not affected in SPG5 cortical neurons, as revealed by the intensity of Filipin staining, whereas immuno-enzymatic quantifications performed via ELISA detected a significant increase of 27-OHC levels. Despite differentiating capacity being indistinguishable from control counterparts, SPG5-derived cortical projection neurons (cortical layer V, stained with CTIP2) exhibited decreased axonal length, determined as limited axonal outgrowth in 36-day-old neurons upon dissociation and replating. In addition, SPG5 mature (3-month-old) cortical projection neurons showed a dramatic increase in neuritic swellings detectable via staining for the Tau protein. Importantly, the treatment of chenodeoxycholic acid (CDCA) demonstrated effectiveness in reducing axonal varicosities of iPS-derived neurons (Figure 2C). CDCA is a natural farnesoid X receptor (FXR) agonist that acts to suppress cholesterol and bile acid biosynthesis through feedback mechanisms and reduces cholesterol accumulation [142,143]. For this reason, CDCA is routinely administered in the clinical treatment of CTX patients to mitigate symptoms and disease progression [144,145]. The findings obtained in that study were also corroborated by the coincident results observed in cortical projection neurons derived from isogenic *CYP7B1* KO human embryonic stem cells, generated via CRISPR-Cas9-mediated genome editing. Overall, SPG5 iPSC-derived neurons proved to be a valid model faithfully recapitulating the hallmarks of HSP and providing a suitable platform for preclinical drug screening. This study showed that CDCA, a metabolic modulator of cholesterol, is a promising tool to counteract axonal neuropathy in SPG5 patients, while also counting the absence of drug tolerability and side effect issues due to common use in CTX management.

### 2.4. SPG7

Spastic paraplegia 7 (SPG7) is one of the most frequent AR-HSPs presenting a complicated phenotype that reproduces the multifaceted phenotype of mitochondrial diseases [146]. The disorder has a general adulthood onset and is characterized by leg weakness, spasticity, and other different manifestations, such as cerebellar signs/cerebellar atrophy, optic neuropathy [147,148,149], hearing loss, ptosis [150,151], and supranuclear palsy [152]. Hypokinetic movement disorders and lower motor neuron features can also be associated with SPG7.

SPG7 is caused by biallelic mutations in the *SPG7* gene, encoding for Paraplegin, a zinc matrix metalloproteinase located in the inner mitochondrial membrane (Figure 1). Paraplegin is involved in multiple processes, such as protein quality control, proteolytic activation of essential mitochondrial proteins, ribosome assembly, and mitochondrial biogenesis. Ultrastructural studies performed in SPG7-derived cells have shown the occurrence of abnormally shaped mitochondria [147,153].

The previously mentioned ONS model proved useful to investigate mitochondrial dysfunction in SPG7 [97]. After staining with MitoTracker Green, SPG7 patient-derived ONS cells displayed short and highly fragmented mitochondria, with a low degree of branching and interconnectivity, which are typical signs of a dysfunctional mitochondrial network. Compared to the controls, SPG7 ONS displayed reduced basal respiration, significantly lower levels of oxygen consumption attributed to ATP production, maximal respiration, and reduced spare respiratory capacity. In addition, live cells were also stained with MitoSOX, a mitochondria-specific superoxide indicator, and CM-H2DCFDA, a general reactive oxygen species indicator. The observed fluorescence signals of both indicators were reported to be significantly higher in *SPG7* mutant cells compared to the control counterparts (Figure 2D). Finally, *SPG7* mutant cells displayed reduced cellular proliferation, in line with previously reported findings on cells carrying mutations in other known mitochondrial disease genes [154]. In addition, to study Paraplegin deficiency directly in cortical neurons, iPS cells were derived from peripheral blood mononuclear cells taken from SPG7 patients carrying different pathogenic variants [155]. Cells were differentiated in cortical progenitors and neurons via classical dual SMAD inhibition [156,157]. Despite the indistinguishable expression of pan-neuronal (beta-3 tubulin) and cortical (TBR1 and CTIP2) markers, neurite length and complexity were consistently reduced in patient-derived mature (30 days in vitro) cortical neurons compared to control cells. Moreover, both SPG7 progenitors and neurons displayed a slight increase in mitochondrial size-related parameters (i.e., whole area, perimeter, length, width), accompanied by a reduction in mitochondrial potential (as assessed via TMRM staining) and a likely correlated diminished cell viability. Transcriptomic analysis showed the downregulation of genes related to synaptic function in SPG7 neurons, and electrophysiological responses of SPG7 cortical neurons were also consistently reduced. As mitochondrial dysfunction was deemed to be the primal cause for all these phenotypes, differentiating SPG7 progenitors were treated with Bz-423, a drug shown to be effective in rescuing mitochondrial and neurological function in an SPG7 mouse model [49] (Table 1). The treatment was able to rescue—almost completely—disease-associated neuronal phenotypes, posing hopes for the development of future therapeutic strategies in SPG7 patients, at least in the early stages of the disease. The same group obtained similar results using genuine patient-derived iPS-differentiated cortical neurons, which display reduced neuritic complexity, mitochondrial dysfunction, and increased degeneration. Also in this case, treatment with Bz-423 was able to rescue most of the phenotypes at nanomolar concentrations, thereby establishing a direct link between mitochondrial and neuronal defects in SPG7 neurons [155]. Unfortunately, Bz-423 induces cytostasis and cytotoxicity at higher concentrations and is immunomodulatory [158], so safer compounds will need to be tested as putative replacements. Still, SPG7 iPS-derived neurons will remain a very useful platform for drug discovery, as all studies discussed above certified these cells as a faithful model for SPG7.

### 2.5. SPG57

Biallelic mutations in TFG (tropomyosin receptor kinase) have been implicated in corticospinal axon pathology, causing neurological disorders, such as ALS, hereditary motor and sensory neuropathies (HMSNs), and Spastic paraplegia 57 (SPG57).

SPG57 presents in fewer families with lower limb spasticity, optic atrophy, and polyneuropathy. Consistently, the protein is highly expressed in mouse Purkinje cells and infragranular cortex, where it localizes either in the soma or axonal and dendritic projections [159,160,161,162].

TFG is a conserved regulator of the early secretory pathway, controlling the export of protein cargoes from ER. The protein multimerizes in cup-like octameric structures that create a meshwork between ER and ER-Golgi intermediate compartment (ERGIC), facilitating interaction between the scaffolding protein SEC-16 and COPII-coated transport carriers (Figure 1). In the absence of TFG, COP-II vesicles tend to scatter away ER exit sites, slowing down vesicle transport and triggering an ER stress response [73,163].

Overexpression of the Arg106Cys variant in cell lines not only disrupts normal ER organization but also alters the distribution of mitochondria, promoting clusters of these organelles around microtubule organizing centers. Despite this, cytoskeletal components themselves do not appear to be affected [160]. In agreement with these results, when overexpressed in mouse primary hippocampal neurons, known TFG pathogenic variants (Arg106Cys/His) dramatically increased mitochondrial fragmentation [164]. CRISPR-Cas9 genome editing in the well-characterized human IMRO90-4 iPS cell lines created an Arg106Cys model of TFG R106C [107]. The derived cortical glutamatergic neurons from control and genome-edited iPS cells revealed no differences in terms of timing of either neuronal differentiation or the emergence of spontaneous electrical activity. Also, the organization of Golgi was unaffected in mutant i-neurons, though the distribution of the variant protein itself was derailed, as staining was more diffuse and poorly localized at the ER/ERGIC interface. Different from findings in cultured mouse neurons, mitochondrial morphology, and motility along neurites were indistinguishable between control and mutant neurons, and there was neither impaired lysosomal function nor altered autophagic behavior. Finally, axons from neurons expressing TFG Arg106Cys did not show axonal swellings, a finding reproduced in other SPG iPS-derived neurons. To address instead the possibility of impaired axonal outgrowth, similar to SPG3A, telencephalic neurospheres were derived from expressing iPS cells. These floating aggregates, typically cultured on low adhesion surfaces, differentiate in neurons and start to send projections upon adhesion on coated coverslips. Strikingly, although no difference was detectable in terms of axonal outgrowth, neurites from SPG57-derived neurons failed to form axonal bundles, unlike what is usually observed in control neurons (Figure 2E). Homotypic axonal fasciculation is mostly due to the presence of transmembrane adhesion molecules that interact with the extracellular matrix with external domains [165]. A deeper investigation clarified that the defects lie in the early secretory pathway and impair trafficking of the adhesion molecule L1CAM, impairing the capacity of axons to organize in fascicles, a key factor for cells with extremely long projections like corticospinal motoneurons. Intriguingly, gene mutations in L1CAM have been associated with hydrocephalus, severe intellectual disability, aphasia, and HSP (SPG1) [166,167,168,169,170]. Induced pluripotent cells from SPG1 patients have been recently generated [89], and the characterization of differentiated cells is in progress. Also in this case, the use of iPS cells allowed phenotypic characterization of HSP neurons, defining axonal fasciculation issues in SPG57 cells and providing a platform for directed or unbiased pharmacological screening.

### 2.6. SPG11

Spastic paraplegia 11 (SPG11) is the most frequent AR-HSP, comprising about 20% of all AR-HSP and up to 45% of AR-HSP with TCC [146,171]. SPG11 patients generally have a complicated phenotype that includes progressive spasticity and weakness of the lower limbs, mild intellectual disability with learning difficulties, cerebellar signs, and peripheral neuropathy, usually associated with thinner corpus callosum at brain MRI [171,172]. It was also hypothesized that the phenotype results from combined degeneration of central and peripheral axons and neuronal loss within cortical, thalamic, and spinal cord regions [173].

**Figure 3:**
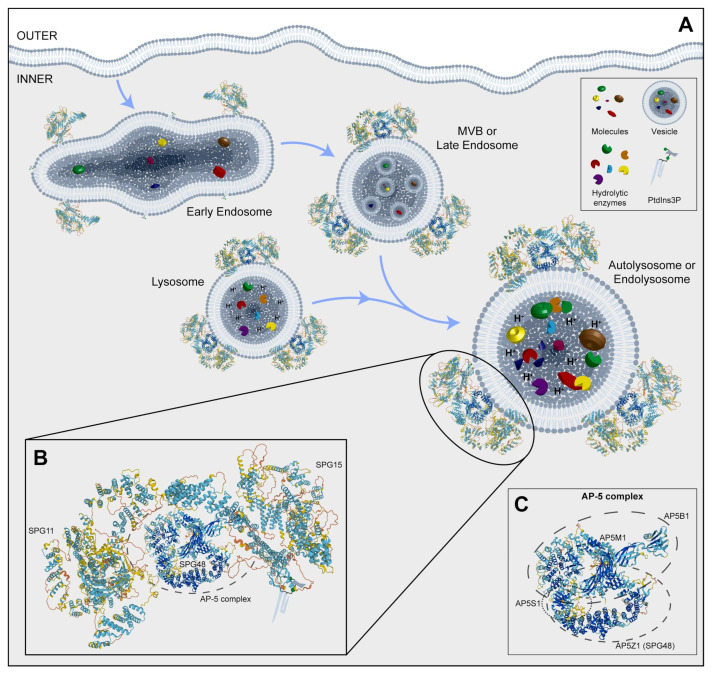
(**A**) Representative image of the endocytosis pathway and localization of SPG11, SPG15, and AP5Z1, which is a member of the AP-5 complex. (**B**) An inset of a possible 3D structure of the SPG11-SPG15-AP-5 complex on vesicles. (**C**) A box with a potential 3D structure of the AP-5 complex and its component four proteins, including SPG48. MVB: multivesicular body; PtdIns3P: phosphatidylinositol 3-phosphate. Protein 3D structures have been generated with Alphafold [118,119].

SPG11 is caused by biallelic mutations in *KIAA1840*, leading to loss of protein function. The *SPG11/KIAA1840* locus spans over 40 exons and encodes for a 2443-amino acid peptide named Spatacsin, a protein expressed ubiquitously in the nervous system, but most prominently in the cerebellum, cerebral cortex, and hippocampus [60,174]. In addition, Spatacsin is expressed throughout neural differentiation and present in dendrites and axons of mouse and human cortical projection neurons, where it associates mostly with synaptosomes. Although the precise function is unknown, Spatacsin has frequently been associated with vesicular trafficking and organelle shaping [3], and it has been associated with autophagy since its pivotal role in autophagic lysosome reformation (ALR) [175] (Figure 3). Downregulation of Spatacsin in vitro was performed via knockdown in primary cortical mouse neurons and the derivation of ES-differentiated cortical neurons with mutations in the SPG11 gene. In order to do this, human iPS cells were derived from three different patients carrying compound heterozygous mutations in SPG11. As controls, iPS cells were derived from two healthy Caucasian individuals with no history of movement disorder or neurological disease.

**Figure 4:**
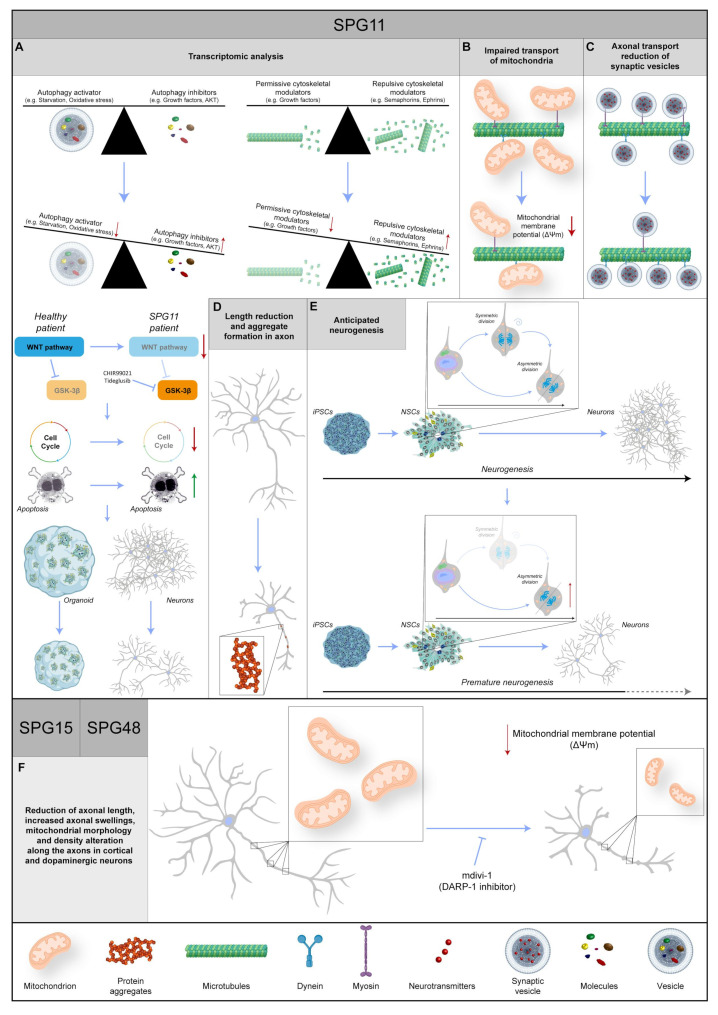
Representative images regarding the known data about three different SPG genes: *SPG11*, *SPG15*, and *SPG48*. (**A**) The transcriptomic analysis shows an increase in repulsive cytoskeletal modulators and autophagy inhibitors in combination with a decrease in permissive cytoskeletal modulators and autophagy activators. Moreover, in the SPG11 patient, the WNT pathway is impaired, leading to a reduction in proliferation and an increase in apoptosis, followed by a decrease in organoid size and the number of neurons. WNT pathway activation via inhibition of GSK-3β with CHIR99021 or Tideglusib rescues the phenotype. (**B**) In SPG11 neurons, the mitochondrial transport is impaired, and their membrane potential is reduced. (**C**) Reduction in synaptic vesicle axonal transport, in particular a decrease in anterograde and an increase in retrograde, is found in SPG11 patients. (**D**) In SPG11 motoneurons, the axons show a length reduction and the formation of aggregates. (**E**) The physiological timing of neurogenesis is disrupted in SPG11 patients, leading to premature differentiation, and resulting in a reduction in neuronal abundance. (**F**) In SPG15 and SPG48 patients, the axons in cortical and dopaminergic neurons show an increase in swelling phenomenon, a length reduction, and alterations in mitochondrial morphology and density. Intriguingly, most phenotypes are rescued with mdivi-1 (DARP-1 inhibitor). Red or green arrows indicate a dysregulation of biochemical pathways, unbalance of protein levels or changes in molecular specie concentrations. Protein 3D structures have been generated with Alphafold [118,119].

Spatacsin-depleted cortical neurons displayed a reduction in anterograde and an increase in retrograde axonal transport, accompanied by a reduction in acetylated tubulin signal, and it strictly correlated to neurite outgrowth impairment and a reduction in their complexity. Electron microscopy analysis of synaptic vesicles in SPG11 neurons demonstrated the presence of a large number of membrane-encircled inclusions within the neuritic compartment, while the expression analysis of transport-related genes pointed out the reduction in transcripts encoding anterograde molecular motors such as *KIF3A*, *KIF5A*, and *KLC1*, as well as tubulin associated genes (*MAP*, *TAU*, and *TTBK1*) and synaptic genes (*VAMP2* and *SYN1*), Interestingly, Spatacsin knockdown not only interfered with axonal growth but also seemed to induce axonal retraction in primary mouse cortical neurons, well-correlating with axonal neuropathy found in SPG11 patients. These results were confirmed by time-lapse experiments studying the trafficking of synaptic vesicles in Synaptophysin-mCherry-transfected SPG11 cortical neurons cultured in microfluidic chambers. Consistently, mutant neurons displayed a significant reduction in anterograde transport, accompanied by an increased number of axonal processes showing no vesicular movement or even increased retrograde transport. Collectively, this scenario describes an efficient axonal transport reduction in SPG11 cortical neurons [55]. The effect of Spatacsin ablation was also studied in SPG11 iPS cell-derived spinal motoneurons [101]. Similar to cortical counterparts, differentiated cells displayed a reduction in the total neurite length, impaired mitochondrial anterograde axonal transport, and the presence of ultrastructural neuritic aggregates (Figure 4A–D). In addition, SPG11 motoneurons showed reduced mitochondrial membrane potential and increased lysosomal accumulation, consistent with SPG11 loss of function in animal models [56,57,176].

Gene ontology analysis of differentially regulated transcripts revealed over-represented biological processes mostly associated with distinct stages of neurodevelopmental pathways, including regulation of neurogenesis, nervous system development, and neuron differentiation. Interestingly, the KEGG pathway representation of individual sets of genes showed a clear downregulation of the Wnt pathway, cell cycle transcripts, and regulators of proliferation such as CCNA1 and CDH1, together with genes related to neuronal morphogenesis (Figure 4A). Mediators of repulsive neuronal interactions such as SEMA3A, EPHB1, PLXNB1, NFIA, and NCAM1 were found to instead be greatly upregulated. Those findings are also important since the impaired development of callosal fibers is a common finding in SPG11 patients [171]. Moreover, the expression of autophagy modulators was impaired, with the downregulation of positive and upregulation of negative regulators. Results from the transcriptomic analysis were corroborated with immunohistochemistry of hiPS-derived neural cells, showing reduced proliferation of neural progenitors and accelerated neurogenesis in the SPG11 background, ultimately leading to a reduction in the neuronal population. The formal demonstration of the involvement of the Wnt pathway was confirmed via the TCF/LEF (T-cell factor/lymphoid enhancer factor) ß-Catenin reporter, showing reduced luciferase activity in SPG11 cells, and definitely established by rescuing the observed cellular phenotype by forced activation of the cascade with CHIR99021 or Tideglusib [101]. Tideglusib is a known chemical inhibitor of GSK3 beta, the kinase that destabilizes ß-Catenin, presenting negative feedback to Wnt pathway activation (Figure 4A). Tideglusib treatment almost completely reverted the accumulation of axonal inclusions in Spatacsin-deficient iPS-derived cortical neurons, rescuing the effect on cell death [177].

Using the same SPG11 hiPS lines, the study on bidimensional culture was also extended to free-floating cerebral organoids, displaying a structural organization typical of developing cortical tissue. SPG11 organoids consistently presented smaller sizes in comparison to the control counterparts, with a significative reduction in the progenitor layer thickness after 9 weeks of culture and an enlarged volume of ventricle lumen. As the number of (neurogenic) vertical divisions outnumber horizontal (proliferative) divisions in the ventricular zone of SPG11 organoids, the phenotype observed seems to be caused by a mechanism of increased neurogenesis. Moreover, the cell cycle appears to be longer in Spatacsin-deficient cortical progenitors (Figure 4E). This collectively results in the generation of a consistently reduced number of cortical neurons in SPG11 cortical aggregates, faithfully mirroring what was previously observed in bidimensional cultures. Again, the modulation of the Wnt pathway with Tideglusib almost completely reverted the detrimental effects caused by the Spatacsin absence observed in SPG11 patient-derived cortical organoids (Figure 4A).

All these studies emphasized the role of Spatacsin in the generation and maintenance of cortical and spinal motoneurons, linking the development and neurodegeneration in SGP11 patients and opening new avenues for putative pharmacological treatments using Tideglusib, an FDA-approved drug for clinical use in dental care.

### 2.7. SPG15 and SPG48

Patients harboring variants in SPG15 or SPG48 forms of AR-HSP share similar clinical phenotypes to SPG11 patients, such as thinner corpus callosum, cognitive impairment, ataxia, cataracts, retinopathy, and early onset parkinsonism [172,178,179].

SPG15 is caused by biallelic mutations in *ZFYVE26*. Findings on brain MRI suggestive of SPG15 may include characteristic signal changes in the periventricular white matter, known as the “ears of the lynx” sign [180]. *ZFYVE26* encodes for Spastizin, a protein described to be involved in endosomal trafficking, autophagy, and cytokinesis [178]. Mutated variants of Spastizin are indeed associated with defective autophagy [181]. On the other hand, SPG48 is a rare HSP presenting lower limb spasticity and associated with urinary incontinence caused by biallelic mutations in *KIAA0415/AP5Z1*. The gene encodes the AP5Z1 putative helicase, localized in both the nucleus and cytoplasm, and is involved in DNA double-strand break repair processes [182].

Spatacsin and Spastizin were shown to associate with the heterotetrameric adaptor protein complex 5 (AP-5), implicated in vesicle formation and sorting, while AP5Z1 protein is a subunit of the complex itself (Figure 3) [183]. Mouse models in which those genes have been knocked out consistently share a common impairment of lysosomal dynamics, with an accumulation of abnormal endolysosomes (Table 1). According to the autosomal recessive nature of *AP5Z1* mutations, fibroblasts derived from SPG48 patients contained no AP5Z1 protein. Interestingly, immunostaining of these cells for LAMP1, a known marker of late endosomes and lysosomes, showed the occurrence of numerous large positive puncta, and an accurate morphological examination performed via electron microscopy showed the presence of multilamellar structures filled with abnormal storage material forming exaggerated whorls, belts of striated material, fingerprint bodies, and some intraluminal vesicles [184]. Notably, similar ultrastructural findings have been reported for several lysosomal storage disorders, including metachromatic leukodystrophy, Fabry disease, mucopolysaccharidoses, Niemann–Pick disease, GM2 gangliosidoses, and neuronal ceroid lipofuscinosis [185,186,187], pointing to the hypothesis that AP-5 impairment leads to lysosomal accumulation. Interestingly, the absence of AP5Z1 in HeLa cells neither affected the total level of Spastizin nor the membrane association of Spatacsin. Conversely, the loss of Spatacsin or Spastizin resulted in decreased AP5Z1, suggesting a tight link between these proteins. As Spastizin and AP5Z1 are particularly enriched in neurons of the CNS [184], iPS cell lines were derived from SPG15 and SPG48 patients [102] in order to characterize SPG-derived differentiated neurons. Pluripotent cells were differentiated into cortical, spinal, and mesencephalic dopaminergic (mDA) neurons using established protocols [141,188,189,190] and characterized for the expression of their relative respective identity markers, such as Tbr1, HoxB4, and tyrosine hydroxylase (TH). Despite no difference being observed between SPG and control cells in terms of differentiation capacity for the different neuronal types, the ability to send projections upon neuronal detachment and replating was impaired in SPG15 and SPG48 cortical and dopaminergic neurons, but not spinal neurons. Analysis of neuritic outgrowth indeed showed that both the average neuritic length and the length of the longest neurite were found to be significantly reduced in patient-derived cells. In addition, the typical pathological hallmark of HSP neurons, namely axonal swelling, was present in 6-week-old SPG cortical neurons, as clearly stated by acetylated tubulin staining (Figure 4F).

A couple of premises have led researchers to pay attention to the mitochondrial structures and functions in SPG15 and SPG48. First, as aforementioned, both mutations can present as hypokinetic movement disorders like SPG11, and dopaminergic neurons seem to be particularly sensitive to the loss of function of mitochondrial proteins, such as PINK-1 and Parkin. Second, Spastizin was found to be localized at the mitochondrial surface [191]. Therefore, patient-derived neurons were stained with MitoTracker CMXRos to perform live imaging of mitochondrial morphology in the neuronal neuritic compartment. This experiment revealed a consistent reduction in the average mitochondrial length in both SPG15 and -48 cortical neurons, together with a reduction in the mitochondrial aspect ratio (defined as length/width) in SPG15 cells, a decreased number of mitochondria per 1 µm of neurite for the SPG48 neurons, and an overall reduction in linear mitochondrial density for both groups. Dopaminergic neurons also showed mitochondrial changes, with alterations in the length and aspect ratio in SPG15 neurons and a reduction in mitochondria along neurites in both SPG15 and SPG48 cell lines. In addition, together with morphology, the mitochondrial function of SPG-derived neurons was also assessed by measuring mitochondrial membrane potential with the fluorescent dye tetramethylrhodamine methyl ester (TMRM), as previously described [192]. These experiments revealed a significant reduction in mitochondrial membrane potential in both Spastizin and AP5Z1-deficient cortical neurons, pointing clearly to a decreased capacity of these cells to produce energy through respiratory pathways. In addition, this condition could facilitate the release of mitochondrial proteins in the cytoplasm, like AIF and cytochrome c, thereby triggering the mitochondrial pathway of the apoptotic mechanism [193]. Staining SPG15 and -48 long-term neuronal cultures with antibodies for activated Caspase-3 actually confirmed this hypothesis, with a dramatic increase in positive cells in the mutant neurons compared to the control counterparts. In the attempt to rescue these phenotypes, SPG neurons were treated with mdivi-1 (mitochondrial division inhibitor 1), an inhibitor of DRP1, a key protein mediating mitochondrial fission, as conducted in PINK-1 mutant mDA neurons [194]. Strikingly, a 48 h treatment with mdivi-1 reverted the reduction in the mitochondrial number in SPG telencephalic neurons, increasing their linear concentration in neurites, restoring their normal morphology, and decreasing apoptosis in telencephalic neurons (Figure 4F). Lentiviral knockdown of DRP1 in the same SPG cortical neurons confirmed a direct involvement of the protein in neuritic defects observed in SPG15 and SPG48 neurons. Notably, the same results obtained in SPG-derived telencephalic neurons were also reproduced with the shRNA-mediated knockdown of *ZFYVE26* and *AP5Z1* in H9 human embryonic stem cell-derived forebrain neurons [102]. These cells also displayed a reduction in neurite outgrowth, and similar to original SPG-derived neurons, the phenotype was rescued by treatment with DRP1 inhibitor mdivi-1.

Therefore, in the case of the SPG11-SPG15-SPG48 protein complex, the iPS cell-derived model offered a powerful tool to study mechanisms, corroborating their role in establishing and maintaining the intricate axonal projections in forebrain glutamatergic neurons. It will indeed be interesting in the future to see the possible utilization of mdivi-1 in clinical trials for these HSP forms.

### 2.8. AP-4 Complex (SPG47, SPG50, SPG51, SPG52)

**Figure 5:**
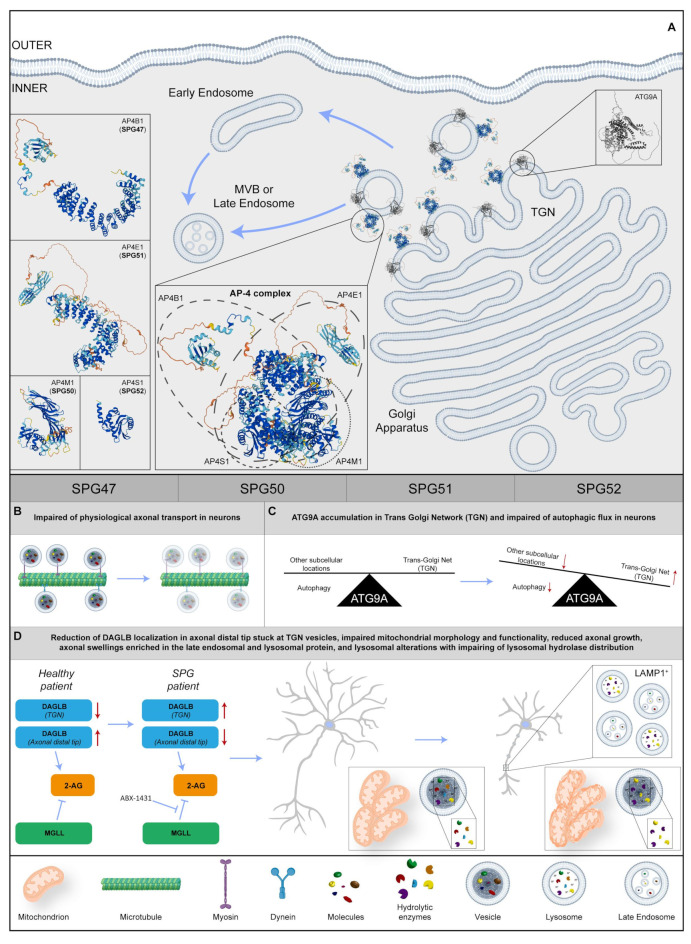
(**A**) Representative image regarding the physiological localization and possible 3D structure of four SPG proteins: SPG47, SPG50, SPG51, and SPG52. In particular, they shape the AP-4 complex at the Trans-Golgi Network (TGN) in the Golgi Apparatus, trafficking in transmembrane proteins from TGN to early or late endosomes. Regarding the insets on the left, 3D structures of each SPG protein are shown and, in the central box, there is a potential 3D structure of the AP-4 complex and the component’s four SPG proteins. (**B**) In the SPG patient, the axonal transport is disrupted in neurons. (**C**) In neurons, ATG9A remains at the TGN level, impairing the physiological autophagy. (**D**) In SPG neurons, DAGLB (diacylglycerol lipase-beta) accumulates in TGN, reducing 2-arachidonoylglycerol (2-AG) production in axonal tips. Inhibition of monoacylglycerol lipase (MGLL) rescues the axonal phenotype. Moreover, the impairment of axonal growth and axonal swelling is found, together with mitochondrial aberration and lysosomal dysfunction. Red arrows indicate a dysregulation of biochemical pathways, unbalance of protein levels or changes in molecular specie concentrations. Protein 3D structures have been generated with Alphafold [118,119].

Adaptor proteins (AP) constitute a conserved family of heterotrimeric protein complexes that work by facilitating the assembly of cargo proteins in vesicles and recruiting all the proteins required for transport and budding [195]. The AP-4 complex is composed of four different subunits, encoded by the genes *AP4B1*, *AP4M1*, *AP4E1*, and *AP4S1* (Figure 5A). Biallelic mutations in these genes lead to typical forms of childhood-onset-complicated HSPs (SPG47, SPG50, SPG51, and SPG52, respectively), together called AP-4 complex-associated HSPs. Clinical manifestations in the AP-4 complex disease include delayed psychomotor development, progressive spasticity beginning in the lower limbs resulting over time in spastic tetraplegia, and intellectual disability with the absence of speech, post-natal microcephaly, and epilepsy [196]. Neuroimaging shows that TCC, nonspecific white matter abnormalities, ventriculomegaly, and colpocephaly can be observed, associated with cortical, cerebellar, and cerebral atrophy in patients with advanced disease.

The AP-4 complex is known to be implicated in the trafficking of transmembrane proteins, from TGN to early- and late-endosomal compartments [197]. Studies in cultured cells revealed that AP-4 is also involved in the transport of ATG9A, an important player in the formation of autophagosomes. The loss of the AP-4 function leads to the lack of the export of ATG9A from TGN, impairing its physiological axonal transport and proper neuronal autophagic flux [198,199] (Figure 5A–C). In line with these findings, the *Ap4e1* knockout mouse displays reduced axonal developmental growth and the occurrence of axonal swellings [70,71].

To broaden the knowledge on AP-4 deficiency in human neurons, dermal fibroblast lines from seven patients carrying biallelic mutations in *AP4B1*, three in *AP4M1*, one in *AP4E1*, and three patients with mutations in *AP4S1*, were studied [105] to investigate levels and localization of ATG9A. The protein increased in patient fibroblasts, and almost completely colocalized with the trans-Golgi marker, TGN46. The rescue of the phenotype with the redistribution of ATG9A to the physiological subcellular localization was conducted by infecting *AP4B1* fibroblasts with the lentiviral vector expressing AP4B1. AP4B1 fibroblasts were also used to derive iPS cells, and in turn, were differentiated in cortical glutamatergic neurons via the overexpression of Neurogenin-2 [200]. Induced neurons were harvested in 96-well plates and images were acquired through automated high-content confocal microscopy. With this approach it was possible to demonstrate that patient-derived i-neurons reproduced TGN accumulation of ATG9A, making this cellular phenotype a likely target for high-content drug screening tools. In addition, i-neurons showed impaired mitochondrial morphology and functionality (Figure 5D). In line with these results, morphological analysis of SPG51 patient-derived cortical neurons showed defective neuritic growth, mimicking in vitro the clinical findings of thin corpus callosum usually found in patients, and corroborating the hypothesis that mutant iPS-derived neurons could represent a faithful in vitro model to study pathological mechanisms of AP-4 HSP.

A more recent study exploited advanced proteomic approaches to search for cargo proteins of the AP-4 vesicle pathway, identifying DAGLB (diacylglycerol lipase-beta) as a novel interactor of the complex [201]. DAGLB is a serine lipase that hydrolyzes diacylglycerol (DAG) for the generation of 2-arachidonoylglycerol (2-AG), the most abundant endocannabinoid in the whole brain. The enzyme is localized at developmental stages in the axonal distal tip [202], where 2-AG is required to activate CB1 and CB2 cannabinoid receptors for axonal growth and guidance, especially for the elongation and fasciculation of the long axons of pyramidal cells [203,204,205]. Similar to what was observed for ATG9A, DAGLB showed a TGN subcellular localization in AP-4-deficient cell lines, and colocalization with the AP-4 accessory protein RUSC2 and cargo proteins SERINC1 and SERINC3 [201]. Most importantly, DAGLB localization was significantly reduced in axonal processes of *AP4B1* mutant iPSC-derived neurons, with most of the protein stuck in TGN vesicles. As a most likely consequence, mutant neurons also showed a reduction in axonal length and branching, reminiscent of axonal pathology classically described in AP-4-HSP patients. As levels of 2-AG showed a considerable decrease in the brains of Ap-4-deficient mice, researchers elaborated a strategy to increase endocannabinoid levels via the chemical inhibition of monoacylglycerol lipase (MGLL, aka MAGL), the enzyme responsible for the hydrolysis of 2-AG into arachidonic acid [206]. Strikingly, treatment with the highly selective MGLL inhibitor (ABX-1431) was sufficient to rescue the axonal phenotype in AP-4-deficient neurons, while leaving control neurons completely unaffected. Taken together, these data strongly support the involvement of 2-AG in AP-4-HSP, opening the door for future therapeutic approaches with inhibitors of endocannabinoid catabolism (Figure 5D).

While autophagy involvement has been extensively studied in the pathophysiology of the AP-4 complex, few studies have characterized possible alterations of the lysosomal compartment in animal and cellular models. Interestingly, axonal swellings in the hippocampus and midbrain of *Ap4e1* KO mice were enriched in the late endosomal and lysosomal protein, LAMP1 [207] (Figure 5D).

To fill this gap, multiple research groups [208,209,210] have made use of a CRISPRi-iPSC system to enable the Neurogenin-2-induced rapid generation of glutamatergic telencephalic neurons from pluripotent stem cells with knockdown of the *AP4E1* gene.

The efficiency of the lysosomal proteolytic function, as assessed by the DQ-Red bovine serum albumin (BSA) trafficking assay, was decreased in mutant neurons, and the distribution of lysosomal hydrolases was also impaired in AP-4-deficient cells, suggesting that the protein complex regulates trafficking of specific lysosomal proteases (Figure 5D). As this process was known to be regulated by receptors such as Sortilin [211], it was possible to define the involvement of the lysosomal dysfunction in the pathogenesis of spastic paraplegia diseases due to AP-4 deficiency, thereby confirming the usefulness of patient-derived iPS cells in the identification of new potential molecular mechanisms.

### 2.9. Other SPG Genes

In addition to the aforementioned examples in common forms of HSP, a series of iPS cell lines have been derived from patients affected by other, relatively less common forms, including SPG1 [89], SPG10 [98], SPG30 [103], SPG43 [104], SPG56 [106], SPG58 [108], and SPG76 [110]. For all of these, extensive studies that characterize the differentiation of pluripotent cells in neuronal populations involved in the disease, such as cerebellar, dopaminergic, spinal, and corticospinal neurons, are needed.

Among them, SPG10, SPG30 and SPG58 are complicated forms of HSP caused by mutations in genes (*KIF5A*, *KIF1A*, and *KIF1C*, respectively) coding for kinesin molecular motors, critical for a proper synaptic function, especially for corticospinal motoneurons typically affected in paraparesis. KIF1A seems to be required for neurofilament transport [212], as well as for synaptic vesicle precursors [213,214] in mouse primary cortical and hippocampal neurons. Thus, the disruption of the kinesin function or even haploinsufficiency (as in SPG10 and SPG30) is sufficient enough to cause spastic symptoms [132,215].

On the other hand, SPG56 patients suffer from early-onset HSP with a wide spectrum of neurodevelopmental neuroimaging manifestations, including basal ganglia calcification, TCC, and hypomyelination [216,217]. The disease has been associated with null mutations in the *CYP2U1* gene, coding for a member of the cytochrome P450 family, well-known for acting in the conversion of natural substrates into locally active signaling molecules. The CYP2U1 protein has been shown to participate in the hydrolyzation of riboflavin (vitamin B2), a flavonoid precursor of active compounds like flavin mononucleotide (FMN) and flavin adenine dinucleotide (FAD), which are crucial for oxidative phosphorylation reactions in mitochondria. In addition, lipidomic studies in Cyp2u1 null brains imply high concentrations of coenzyme Q10 in patients. These data suggest that the reduction in ubiquinol in ubiquinone exerted by the FAD/FMN-dependent CoQ oxidoreductases might not work properly in SPG56 patients, with the obvious consequence of impaired cellular respiration particularly affecting sensitive cellular populations, such as corticospinal motoneurons and cone photoreceptors [217].

Finally, another example is SPG83, causing symptoms ranging from microcephaly and severe intellectual disability to pure late-onset HSP caused by biallelic variants in *HPDL* [218,219,220]. The gene encodes for an enzyme that was recently associated with an alternative pathway to coenzyme Q10 biosynthesis [221]. Our laboratory is directly involved in the derivation of several patient-derived pluripotent cell lines for the latter two forms.

## 3. Conclusions with a Look over the Horizon

The panorama of HSP patient-derived iPS cells is rapidly expanding, with a growing amount of literature describing in vitro systems as valid models to study the neurophysiology of SPG neurons at the cellular level. The level of faithfulness of the iPS cellular model is increasing, considering the technological breakthrough of modern cell biology, which allowed the assessment of SPG etiopathogenesis in 3D neural structures, from brain organoids to even more complex systems, such as cortico-spinal or cortico-motor assembloids [222,223]. These recent models are capable of entirely reconstructing, in vitro, the motor pathways typically hit in HSPs, starting from the cortex to the pyramidal tract, passing through the spinal cord, and ending in the final target, the skeletal muscle.

Despite having great potential, such iPS-derived neural aggregates remain reductionistic models compared to the overall complexity of both the brain and the entire organism. For instance, some brain cellular populations with crucial roles in triggering and spreading neurodegenerative diseases, such as microglia, vascular cells, and pericytes (reviewed in [224,225]), have a different developmental origin from the neural tube and are, therefore, not normally produced in brain organoids. Integration of those cells into cortico-motor assembloids, similar to what was recently achieved in several labs (reviewed in [226,227]), could partially fill these known gaps. However, increasing the cellular complexity of the system may not be enough to avoid limitations associated with the use of iPS models for the study of neurodegenerative diseases.

From the metabolic point of view, the procedure of reprogramming itself causes a tremendous switch to high-glycolytic and low-OXPHOS fluxes for ATP production in pluripotent cells compared to somatic cells, with a consequent increase in intermediate metabolites exerting a modification of the transcriptional landscape. The reprogramming process also acts on mitochondrial morphology, causing remodeling from an elongated interlacing network characteristic of somatic cells to a fragmented collection of round-shaped and small-sized organelles (reviewed in [228]). These profound modifications should be carefully taken into account, especially in cells carrying mitochondrial defects such as the ones derived from many SPG patients.

Another common issue encountered in iPS research that should be carefully taken into account is the known variability among different pluripotent clones generated from the same donor, especially with the obvious perspective of making comparisons with wild type pluripotent cell lines [229,230]. This limit has been classically solved either by analyzing two or more clones from the same donor or via the generation of isogenic lines in which the mutation has been corrected via homologous recombination or genome editing strategies such as CRISPR-Cas9 or base editing [231,232].

The rise in iPS cells triggered hope in the field of regenerative medicine, and HSP patients could potentially benefit from a cell replacement therapy based on differentiated cell populations as corticospinal motoneurons derived from mutation-corrected progenitors. Several clinical trials are ongoing for the treatment of diverse neurodegenerative diseases, such as macular degeneration [233], Parkinson’s disease [234], and others. Even if, to date, preliminary results indicate safety for their use in both allogeneic and autologous transplantation, the urgency to switch from culture methods used in basic research to cGMP-clinically adapted protocols is still present due to the known tumorigenic capacity in recipients from potentially contaminating residual pluripotent cells in the graft preparation. Rigorous cell selection procedures, the use of xeno-free reagents, and strict QC testing will be necessary to mitigate these risks and, therefore, translate these promising models from bench to bedside [235].

Deep analysis of transcriptional and electrophysiological profiles of iPS-derived neurons displays a trend to have more closely resembling primary fetal brain cells rather than adult neurons [236,237].

Despite this, iPS-derived neurons were reported to show disease-associated characteristics even for adult-onset disorders [238] and associated references, thereby offering an unprecedented opportunity to model neurodegenerative diseases and a platform for drug discovery.

Based on these assumptions, it is plausible for iPS cells to disclose—in the near future—an unmatched view for the study of HSP diseases, for both basic research and as a platform in high-throughput preclinical pharmacological screening, as previously conducted in a number of studies for Alzheimer’s disease, fragile X syndrome, Parkinson’s disease, amyotrophic lateral sclerosis, Friedreich’s ataxia, and others (see Vandana et al., 2023 [239] and references therein).

With the final goal of personalized medicine, we can anticipate that these models will provide patient-specific “avatars”, partially substituting needs for animal models, given ethical constraints regarding the use of vertebrate models in medical research. In this way, the use of patient-derived iPS cells will make it possible to integrate new and old findings in an exhaustive scenario, predict HSP courses in different genomic backgrounds, and define specific therapeutic plans for every SPG patient.

## Figures and Tables

**Table 1:** HSP and related animal models. Published animal models for SPGs related to cellular models discussed in this review are listed in this table. OE, over-expression; KD, knockdown; KO, knock-out; IUE, in utero electroporation; CNS, central nervous system; KI, knock-in; AAV, adeno-associated virus; MO, morpholino.

HSP Designation	Human Gene	Protein Name	Animal Model	Gene Modification	Main Phenotype	References
**SPG3A**	***ATL1***	**ATLASTIN1**	Mouse	KD in primary cortical neurons	Impairment of axonal growth and dendritic branching defects	[19]
				OE in cortical upper layer neurons (IUE) of WT and R217Q Atlastin1	Increase in the formation and/or maintenance of new segments within existing dendritic trees in neurons by WT but not mutant variant. No effect on radial cortical migration.	[20]
			Rat	Expression of ATL1 R217Q mutant (Primary hippocampal neurons)	Impaired protein synthesis. Reduced dendritic spine density	[21]
			Danio rerio	Morphant	Significant reduction of larval motility, with an increase of axonal branching in spinal motoneurons. Phenotype specific to motor and cerebellar neurons.	[22,23]
				OE (human and zebrafish mRNA)	Complete loss of ventral structures likely via inhibition of BMP signaling	[22,23]
			Drosophila melanogaster	KO	High mortality. Small size. Age-dependent locomotor deficits due to degeneration of dopaminergic neurons. Rescued by levo-dopa and SK&F 38393 (dopamine agonists)	[24]
				KO	Reduction of muscle size, increase of synaptic and satellite boutons in motor neurons	[25]
				KD and OE in motor neurons	Larvae: decreased crawling speed and contraction frequency; Adult flies: age-dependent degeneration of dopaminergic neurons, decline in climbing ability	[26]
				OE of pathological variants (R214C, C350R, M383T, R192Q)	Lethality at larval stage and eye size reduction. Heavy overfusion of ER membranes in neuronal cytoplasm. Phenotype severity is dependent on the % of activity of the protein variant (R214C > C350R > M383T > R192Q, with the last having almost no phenotype).	[27]
				CRISPR-Cas9 mediated KI of pathological variants (R214C, C350R, M383T, R192Q)	Decrease of the adult eclosion rate and a reduction in size of all developmental stages. Phenotype severity is dependent on the % of activity of the protein variant (R192Q > M383T > C350R > R214C, with the last having almost no phenotype).	[27]
				KD	Embryonic lethality, reduced lifespan of surviving animals. Eye size reduction. ER reduction and fragmentation in neurons. No impairment of secretory traffic	[28]
				Eye/neural ganglia specific OE of WT Atlastin	Eye size reduction	[28]
				Motoneuron specific OE of WT Atlastin	Expansion of ER membranes, severe impairment of secretory traffic (absence of Golgi complex)	[28]
				OE of mutant K51A variant (different drivers)	Normal eyes and fly survival	[28]
				Motoneuron specific KO	Increases synaptic and satellite boutons in the same way that constitutively activating the BMP-receptor Tkv (thick veins).	[29]
**SPG4**	***SPAST***	**SPASTIN**	Mouse	KO (SpΔ/Δ)	Late and mild motor defect for progressive CNS axonal degeneration. Presence of focal axonal swellings, associated with abnormal accumulation of organelles and cytoskeletal components. Focal impairment of retrograde transport	[30]
				KO (Spast^ΔE7/ΔE7^)	Gait abnormalities.Axonal swellings and impaired anterograde transport in primary cortical neurons. Late and mild motor defect for progressive CNS axonal degeneration. transport and outgrowth deficits accompanied by reduced motor control. Deficits in working and associative memory	[31]
				KO (Sp^Δ/Δ^; Primary cortical neurons)	Axonal swellings and cargo stalling, likely due to impairment of microtubule dynamics. Phenotype rescued by microtubule-targeting drugs	[32]
				KI (ROSA26R-hSPAST-C448Y)	Adult-onset gait deficiencies and corticospinal degeneration. No axonal swellings	[33]
				Spast KO X Spast KI (ROSA26R-hSPAST-C448Y)	Same features as the hSPASTC448Y mouse, but with axonal swellings and stronger and earlier onset	[33]
			Rat	KD in hippocampal neurons	Reduction of axonal branches and overall length	[34]
				OE of WT Spastin in hippocampal neurons	Increase in axonal branches (mostly minor processes)	[34]
				OE of human M1-C448Y and M87-C448Y variants in cortical neurons	Decreased ratio of cells with long processes, increased ratio of cells with short processes. M1-C448Y decreased outgrowth	[35]
				OE of M1 N184X, M1 S245X, or M87 S245X truncated variants in primary cortical neurons	Decreased neuritic outgrowth	[36]
			Danio rerio	Morphant	Impairment of branchiomotor and spinal mototoneuron axonal outhgrowth. CNS-specific apoptosis. Motility defects. Phenotype is not rescued by nocodazole	[37,38]
				Morphant (Spast X Protrudin)	Severe impairment of spinal and branchiomotor axonal outgrowth. Rescued by OE of human Spastin and Protrudin	[39]
				Morphants (specific for DrM1 or DrM61 isoforms)	Curved-tail phenotype and yolk tube agenesis in KD of M1 isoform. Smaller eyes in KD of M61 isoform. Isoform-specific motor neuron and locomotion defects, not rescued by the selective expression of the other isoform.	[23]
				Morphant	Aberrant branching and truncation of spinal motor axons, associated with dysregulated endosomal tubulation	[40]
				Morphant	Hydrocephalia, perturbed yolk sac extension, arched-back phenotype, and high oxydative stress. Partially rescued by phenazine, methylene blue, guanabenz and salubrinal	[41]
				KO (CRISPant)	Impairment of metabolic properties: reduction of mitochondrial respiration in larvae; reduction size, weight and BMI in adults. Impairment of sarcoplasmic reticulum in skeletal muscles	[42]
			C. elegans	KD	Progressive motor defects, reduced lifespan, ER stress and increased oxydative stress. Rescued by guanabenz, salubrinal, phenazine, and methylene blue	[41]
			Drosophila melanogaster	KO (spastin^5.75^)	Severe movement defects. Larval neuromuscular junction (NMJ) phenotypes (boutons are smaller, more numerous and clustered; synaptic transmission is impaired)	[43]
				Neuronal-specific OE	Embryonic CNS collapse	[43]
				KD (ubiquitous)	Lethal in embryonic or early larval stage	[44]
				OE (ubiquitous)	Lethal in embryonic or early larval stage	[44]
				Neuronal KD	Severely impaired adult locomotor performance, apoptosis, decreased lifespan. Partial rescue by treatment with vinblastine	[44,45]
				Neuronal OE (Dspastin K467R)	Severely impaired adult locomotor performance, apoptosis, decreased lifespan. Partial rescue by treatment with vinblastine	[44,45]
				Neuronal OE of human M1-C448Y and M87-C448Y variants	Mutants M1 *spast* showed a more severe phenotype than mutant M87 with a high axonal degeneration in the corticospinal tracts.	[35]
				Neuronal-specific KD	Severe climbing defects and ER stress. Rescued by methylene blue, phenazine, and N-acetyl-L-cysteine	[41]
				KO (spastin^5.75^) X Glial KD of Pak3	No phenotype observed (glial involvement in spastin phenotype)	[46]
**SPG7**	***SPG7***	**PARAPLEGIN**	Mouse	KO	Spastic-ataxic symptoms associated to distal axonopathy of spinal and peripheral axons, characterized by axonal swelling and degeneration due to accumulation of organelles and neurofilaments. Rescued with intramuscular injection of AAV-Paraplegin	[47,48]
				KO (cortical neurons)	Impaired mitochondrial permeability and altered electrophysiological patterns. Rescued by treatment with Bz-423	[49]
				KO	Partial rescue of motor phenotype by Bz-423 administration	[49]
				Spg7 X Afg3l2 double mutant	Early-onset spastic phenotye, complicated with cerebellar ataxia (closer to human disease)	[50]
				eSpg7 KO (Spg7 X Afgrl1 double mutant)	Progressive motor phenotype with uncoordinated gait, and degeneration not only of long spinal axons, but also of spinocerebellar and cerebellar granule axons	[51]
			Drosophila melanogaster	KO	Reduced lifespan. Progressive loss of ability to control movements, greater sensitivity to chemical and environmental stress, muscular and neuronal degeneration. Reduced activity of respiratory chain complexes I and II, severely swollen and aberrantly shaped mitochondria in the synaptic terminals of photoreceptors.	[52]
**SPG5A**	***CYP7B1***	**CYP7B1**	Mouse	KO	Accumulation of 25- and 27-hydroxycholesterol in plasma and tissues, normal levels of brain-derived 24(S)-hydroxycholesterol. No recapitulation of human disease: normal neuronal morphology, no neurological defects, no motor defect, no learning and memory capacities.	[53,54]
**SPG11**	***SPG11/KIAA1840***	**SPATACSIN**	Mouse	KD (Primary cortical neurons)	Reduction of axonal outgrowth	[55]
				KO	Spastic paraplegia and progressive loss of cortical motoneurons and Purkinje cells for accumulation of autolysosome-derived material. No motor or cognitive deficits, amyotrophy or alterations of the corpus callosum	[56]
				KO (stop codons exon 32)	Early-onset motor and cognitive deficit. Cortical, cerebellar, hippocampal and spinal cord atrophy. Corticospinal tract degeneration. Accumulation of gangliosides in lysosomes. Partial rescue in primary neurons by miglustat	[57,58]
				KO (primary cortical neurons)	Decreased number of tubular lysosomes in axons	[59]
			Danio rerio	Morphant	Abnormal twisted/bent tail phenotype. Mild hydrocephaly, small eyes, generalized perturbation of neuronal differentiation. Irregular motor neuron outgrowth	[60]
				Morphant	Abnormal twisted/bent tail phenotype. No major brain abnormalities. Reduction of larval motility.	[61]
				Morphant	Loss of motility or paralysis accompanied by accumulation of GM2. Partial rescue by miglustat	[58]
**SPG15**	***ZFYVE26***	**SPASTIZIN**	Mouse	KO	Late-onset spastic paraplegia with cerebellar ataxia due to loss of both cortical motoneurons and Purkinje cells. High levels of lysosomal enzymes in brain of aged mice (lysosomal dysfunction)	[62]
				zfyve26 X spg11 double KO	Simultaneous disruption does not aggravate the phenotype	[63]
				KO (primary cortical neurons)	Defective anterograde transport, impaired neurite outgrowth, axonal swelling, reduced autophagic flux, lipid accumulation within the lysosomal compartment. Synaptic dysfunction, accompanied by augmented vulnerability to glutamate-induced excitotoxicity	[64]
			Danio rerio	Morphant	Severely curly tails. Reduction of head and eye volume. Motor deficits. Impaired spinal motor neuron axon outgrowth and neuromuscular junction connections	[61]
			Drosophila melanogaster	KO	Reduced lifespan, locomotor deficits. Autophagosome accumulation, enlarged lysosomes, reduced free lysosomes, autophagic lysosome reformation defects. Small-Molecule Enhancer of Rapamycin 28 rescues both autophagic lysosome reformation defects and locomotor deficit.	[65]
**SPG48**	***AP5Z1***	**AP5Z1**	Mouse	KO	Age-dependent degeneration of corticospinal axons due to a block of autophagic flux. Late onset progressive gait abnormalities (recapitulates human phenotype)	[66]
**SPG47**	***AP4B1***	**AP4B1**	Mouse	KO	Thin corpus callosum, enlarged lateral ventricles, motor co-ordination deficits, hyperactivity, a hindlimb clasping phenotype, and neurodegeneration. Accumulation of AMPA receptors in autophagosomes in axons of Purkinje cells and hippocampal neurons	[67,68]
			Danio rerio	CRISPant	Microcephaly, neural necrosis. Alteration of expression level and pattern of autophagy genes atg9a and map1lc3b.	[69]
**SPG50**	***AP4M1***	**AP4M1**	Danio rerio	CRISPant	Microcephaly, neural necrosis. Alteration of expression level and pattern of autophagy genes atg9a and map1lc3b.	[69]
**SPG51**	***AP4E1***	**AP4E1**	Mouse	KO	Hindlimb clasping, decreased motor coordination and weak grip strength. Thin corpus callosum, lateral ventricle enlargement. Defective autophagosome maturation in leading to axonal swellings and reduced elongation. in various areas of the brain and spinal cord.	[70,71]
			Danio rerio	CRISPant	Microcephaly, neural necrosis. Alteration of expression level and pattern of autophagy genes atg9a and map1lc3b.	[69]
**SPG52**	***AP4S1***	**AP4S1**	Danio rerio	Morphant	Reduction of head size, altered CNS development, locomotor deficits, and abnormal neuronal excitability. Shorter axonal length in spinal cord neurons	[72]
				CRISPant	Microcephaly, neural necrosis. Alteration of expression level and pattern of autophagy genes atg9a and map1lc3b.	[69]
**SPG57**	***TFG***	**TFG**	*C. elegans*	KD	Impaired protein secretion for defects in assembly of complexes containing SEC-16 and COPII proteins	[73]
			Mouse	β-cell specific KO	Marked glucose intolerance with reduced insulin secretion, corrlated with smaller β-cell islets	[74]
				Spinal motoneuron specific KO	Deterioration of motor function and muscle atrophy due to denervation of neuromuscular junctions (NMJs)	[75]
				Muscle specific KO	Normal motor functions, no muscle atrophy. Possible phenotype in aging	[75]
				OE of pathological p.G269V or p.P265A variants in primary cortical neurons	Slightly reduced neurite length and an increased apoptotic cell ratio upon OE of p.P265A variant	[76]
				KD in cortical neurons	Late neurite degeneration and neuronal apoptosis	[76]
			Rat	KI for p.R106C mutation (CRISPR-Cas9 mediated)	Progressive motor deficits, thinning of the corpus callosum, ventriculomegaly, and hind limb spasticity. Slowed trafficking of integral membrane proteins. No lysosomal defects.	[77]
			Danio rerio	Translation blocking morphant (ATG-MO) and splice-blocking morphant (E3I3-MO)	Malformations with a curved body axis, reduced motor capacity, high mortality, significant increase in neuronal apoptosic cells in both brain and spinal cord	[76]

**Table 2:** HSPs and related iPS cells. Published iPS cell models for HSPs are listed in this table. ID, intellectual disability; DD, developmental delay; AD, autosomal dominant; AR, autosomal recessive; KD, knockdown.

HSP Designation	Gene	Protein Name	Type of HSP	Onset	Distinguishing Clinical Features	Frequency	Inheritance	iPSCs/hES-Experiments Done	References
SPG1	*L1CAM*	L1CAM	Complicated	Infancy	ID, Adducted thumb, Corpus callosum hypoplasia, Aphasia, Obstructive hydrocephalus	Rare	X-Linked	Generation of L1CAM shRNA depleted hES cells	[88]
								Generation of iPS model	[89]
SPG3A	*ATL1*	ATLASTIN1	Pure	Infantile to childhood (rarely adult onset)	Very slow progression. May present as spastic diplegic cerebral palsy. Complicated phenotype with peripheral neuropathy or autonomic failure reported	80% of early-onset AD HSP, 10–15% of all AD HSP	AD	Differentiation of forebrain neurons	[90]
						AR inheritance is very rare	AR	Differentiation of lower motoneurons and in vitro neuromuscular junction	[91]
SPG4	*SPAST*	SPASTIN	Pure	Variable from infancy to 7th decade	Cognitive decline & dementia common, Distal amyotrophy variably present, Complicated phenotype with ataxia variably present	40% of AD HSP	AD	Neurons differentiated from Olfactory neurosphere-derived stem cells taken from patients	[92]
								Differentiation of cortical neurons; SPAST KD in hES	[93]
								Differentiation of neurons (putative cortical)	[94]
								Neurons differentiated from Olfactory neurosphere-derived stem cells taken from patients	[95]
								Differentiation of lower motoneurons and in vitro neuromuscular junction	[91]
SPG5	*CYP7B1*	CYP7B1	Pure or complicated	Juvenile to early adulthood	Ataxia, Polyneuropathy, Extrapyramidal signs, MRI signs of leukodystrophy	9 of 172 families with AR HSP	AR	Differentiation of forebrain glutamatergic neurons from patient derived and CRISPR-Cas9 mediated KO	[96]
SPG7	*SPG7*	PARAPLEGIN	Pure or complicated	Juvenile or adulthood	Dysarthria, Ataxia, Optic atrophy, Supranuclear palsy, Mitochondrial abnormalities on skeletal muscle biopsy	5–12% of AR HSP. AD inheritance suggested for some pathogenic variants	AR	Olfactory neurosphere-derived stem cells taken from patients	[97]
SPG10	*KIF5A*	KINESIN5A	Complicated	Juvenile or adulthood	Polyneuropathy; Pes cavus	1–2% of all AD HSP. 5–8% of all complicated AD HSP	AD	Generation of iPS model	[98]
SPG11	*SPG11/KIAA1840*	SPATACSIN	Complicated	Childhood or early adulthood	DD, Optic atrophy, Ataxia, Pseudobulbar signs, Polyneuropathy, Levodopa-responsive parkinsonism, Hypoplastic or absent corpus callosum	5% of AR HSP; 75% of HSP with developmental delay and hypoplasia of corpus callosum	AR	Neurons differentiated from SPG11 hiPS cells	[55]
								Cortical neurons differentiated from SPG11 hiPS cells	[99]
								Human SPG11 cerebral organoids	[100]
								Motoneurons differentiated from SPG11 hiPS cells	[101]
SPG15	*ZFYVE26*	SPASTIZIN	Complicated	Childhood or early adulthood	DD, Optic atrophy, Ataxia, Pseudobulbar signs, Central retinal degeneration, Polyneuropathy	1–2% of AR HSP	AR	Cortical neurons, spinal MNs and DA neurons differentiated from SPG15 iPS	[102]
SPG30	*KIF1A*	KINESIN1A	Pure (for AD inheritance)	Juvenile to adulthood	Mild ID in some individuals. Optic nerve atrophy. Epilepsy (rare)	5–6% of all AD HSP	AD	Generation of iPS model	[103]
SPG43	*C19orf12*		Complicated	Childhood	Amyotrophy, Dysarthria, Multiple contractures, Neurodegeneration with brain iron accumulation	Rare	AR	Generation of iPS model	[104]
SPG48	*AP5Z1*	AP5Z1	Pure	Typically adulthood; rarely infancy	Urinary incontinence, Parkinsonism, Dystonia, Thin corpus callosum, Leukodystrophy, Severe DD in infantile onset	Single family	AR	Cortical neurons, spinal MNs and DA neurons differentiated from SPG48 iPS	[102]
SPG47	*AP4B1*	AP4B1	Complicated	Infancy	Severe ID, Facial dysmorphism, Seizures, Stereotypic laughter w/tongue protrusion	Rare	AR	Cortical neurons differentiated from iPS lines with loss of function of several AP4 complex subunit	[105]
SPG50	*AP4M1*	AP4M1							
SPG51	*AP4E1*	AP4E1							
SPG52	*AP4S1*	AP4S1							
SPG56	*CYP2U1*	CYP2U1	Complicated	Infancy	Severe DD, Dystonia, Polyneuropathy, Calcification of basal ganglia	Rare	AR	Generation of iPS model	[106]
SPG57	*TFG*	TFG	Complicated	Childhood	Optic atrophy, Severe polyneuropathy	Rare	AR	Knock-in hES cells for mutant form of TFG. Differentiation of cortical neurons	[107]
SPG58	*KIF1C*	KINESIN1C	Complicated	Childhood	Spastic ataxia, Dystonia	Rare	AR	Generation of iPS model	[108]
SPG59	*USP8*	USP8	Pure	Childhood	Lower limb spasticity, borderline intellectual disability. Nystagmus and pes equinovarus	Rare	AR	USP8 KO murine and human ES cells	[109]
SPG76	*CAPN1*	CALPAIN 1	Complicated	Young adulthood	Spasticity of the lower limbs. Cerebellar ataxia. Upper limb involvement, foot deformities, dysarthria. Cognition unaffected	Extremely rare	AR	Generation of iPS model	[110]

**Table 3:** Genomic information of relevant HSP genes. HSP genes treated in this review are listed with genomic and RefSeq information taken from NCBI (predicted transcripts were excluded from the table).

HSP Designation	Gene	Gene ID	Genomic Location	RefSeq Transcripts
SPG1	*L1CAM*	3897	chrX:153861516-153888990	4 transcript variants (NM_000425.5; NM_024003.3; NM_001143963.2; NM_001278116.2)
SPG3A	*ATL1*	51062	chr14:50560145-50633045	3 transcript variants (NM_015915.5; NM_181598.4; NM_001127713.1)
SPG4	*SPAST*	6683	chr2:32063556-32157637	5 transcript variants (NM_014946.4; NM_199436.2; NM_001363823.2; NM_001363875.2; NM_001377959.1)
SPG5	*CYP7B1*	9420	chr8:64590851-64798737	2 transcript variants (NM_004820.5; NM_001324112.2)
SPG7	*SPG7*	6687	chr16:89508403-89557766	3 transcript variants (NM_003119.4; NM_199367.3; NM_001363850.1)
SPG10	*KIF5A*	3798	chr12:57550044-57586633	2 transcript variants (NM_004984.4; NM_001354705.2)
SPG11	*SPG11/KIAA1840*	80208	chr15:44562696-44663662	3 transcript variants (NM_025137.4; NM_001160227.2; NM_001411132.1)
SPG15	*ZFYVE26*	23503	chr14:67746522-67816590	1 transcript variant (NM_015346.4)
SPG30	*KIF1A*	547	chr2:240713767-240820219	23 transcript variants (NM_001244008.2; NM_004321.8; NM_001330289.2; NM_001330290.2; NM_001379631.1; NM_001379632.1; NM_001379633.1; NM_001379634.1; NM_001379635.1; NM_001379636.1; NM_001379637.1; NM_001379638.1; NM_001379639.1; NM_001379640.1; NM_001379641.1; NM_001379642.1; NM_001379645.1; NM_001379646.1; NM_001379648.1; NM_001379649.1; NM_001379650.1; NM_001379651.1; NM_001379653.1)
SPG43	*C19orf12*	83636	chr19:29698937-29715261	7 transcript variants (NM_001031726.4; NM_031448.6; NM_001256046.3; NM_001256047.2; NM_001282929.1; NM_001282930.3; NM_001282931.3)
SPG48	*AP5Z1*	9907	chr7:4775623-4794397	3 transcript variants (NM_014855.3; NM_001364858.1; NR_157345.1)
SPG47	*AP4B1*	10717	chr1:113894194-113904799	4 transcript variants (NM_006594.5; NM_001253852.3; NM_001253853.3; NM_001308312.2)
SPG50	*AP4M1*	9179	chr7:100101643-100109039	1 transcript variant (NM_001363671.2)
SPG51	*AP4E1*	23431	chr15:50908683-51005895	2 transcript variants (NM_007347.5; NM_001252127.2)
SPG52	*AP4S1*	11154	chr14:31025649-31096450	6 transcript variants (NM_007077.5 5; NM_001128126.3; NM_001254726.2; NM_001254727.2; NM_001254728.2; NM_001254729.2)
SPG56	*CYP2U1*	113612	chr4:107931549-107953461	1 transcript variant (NM_183075.3)
SPG57	*TFG*	10342	chr3:100709494-100748964	4 transcript variants (NM_006070.6; NM_001007565.2; NM_001195478.2; NM_001195479.2)
SPG58	*KIF1C*	10749	chr17:4997950-5028401	1 transcript variant (NM_006612.6)
SPG59	*USP8*	9101	chr15:50424405-50514421	3 transcript variants (NM_005154.5; NM_001128610.3; NM_001283049.2)
SPG76	*CAPN1*	823	chr11:65181928-65212006	4 transcript variants (NM_001198868.2; NM_005186.4; NM_001198869.2; NR_040008.2)
